# On the Nature and Treatment of Cholera

**Published:** 1855-06

**Authors:** Henry Hartshorne


### MEDICAL EXAMINER.

NEW SERIES.—NO. CXXVI.—JUNE, 1855.

---

### ORIGINAL COMMUNICATIONS.

---

### *On the Nature and Treatment of Cholera.*

By Henry Hartshorne, M. D.

There is reason to believe, with Favell, that the cholera-attack may be, in general, resolved into three grades or stages:

1st. That in which the morbid (unknown, but supposed material) cause has reached only the alimentary canal, or the ganglionic centres which control its movements and secretions; giving rise to the premonitory symptoms, or those of *cholerine.*

2d. That in which the "poison" affects all the ganglionic centres* (miscalled those of the sympathetic nerve), and all the muscles of organic life, as well as some of the voluntary muscles; including in the former category the muscular coat of the stomach and intestines, the heart, bladder, smooth muscular fibres of the bronchial ramules, and the muscular coat of all the arteries and of some of the veins; producing what Prof. Meigs has most graphically called the *cholera squeeze*, or universal tonic spasm of the organic muscular tissue; the pressure of which upon the blood produces an arrest of circulation, and an actual expression or forcible filtration of serum through the coats of the vessels, (and thence out by the mucous membranes), just as urine is filtered from the *corpora Malpighiana* into the uriniferous tubules of the kidney—all those morbid appearances after death,

* Loder, of Moscow, Orton, Delpech, Lizars, Coste, Favell, &c.

which some have called inflammation, being, in truth, merely venous congestion, and the effects of what may be properly called vascular spasm.* It may be noticed, also, that the abundance of *free epithelial cells* in the intestines, after death, upon which so much stress was laid by Prof. Horner and by Böhm, has been proved by Drs. Parkes, Gull, Lindsay, &c., to be the result of maceration and not pathological.†

The pathology of the 3d stage consists in the confirmed poisoning of the *blood*, putting it, in many instances, beyond the power of recovery, so that patients often die after reaction from the collapse, with symptoms analogous to those of low fever. Examples of this occur everywhere, during the prevalence of a malignant epidemic,—as in the Philadelphia Alms House in 1849, and at Columbia, in 1854.

Indications for a rational treatment may, it appears to the writer, be deduced from such a pathology.

In the first stage, mild anodynes and diffusible stimulants, (perhaps, even, certain astringents), are very often sufficient to relieve and check the attack.

Some cases, however, pass directly, without any premonitory diarrhœa, into the collapse.‡ In anticipation of this, when threatening to occur, camphor, opium, chloroform, ammonia, creasote, and the essential oils, have been, as I believe, found to be the best of remedies.

In the second stage, Duchaussoy and Vernois§ have proved the absolute *non-absorption* of medicines. Stomachic stimulants and anodynes, to act locally,—and *ice*, to relieve the agonizing thirst, are all that it is worth while to give. In the early, or merely impending collapse, the external application of heat, as by the hot bath, &c., may do great good; later, it annoys and distresses without advantage, as Legroux and others have shewn.|| I have seen this myself. Mustard cataplasms, according to general report, do more positive good; and so do frictions with red pepper and spirits, linimentum cantharidis, &c.,

* Marsden on Cholera, p. 34.
† Begbie, Edin. Med. and Surg. Journal, Jan. 1855.
‡ Vide Milroy on Cholera and Quarantine, p. 10.
§ Gazette Hebdomadaire, Sept. 8, 1854.
|| Bull. Gén. de Thérapeut., Sept. 15, 1854.

perseveringly employed. The galvanic battery would seem to be a rational means of exciting reaction; it has occasionally succeeded, but not often.* Venous injection has frequently been able to reanimate the patient for a period, and, in a number of cases, has effected final restoration. This must be the *only mode*, unless by inhalation, in which we can affect the system generally in the collapse of cholera. Dr. Little, in 1832 and 1849, introduced small quantities of alcohol in this manner, with perfect success in several instances.† Messrs. Duchaussoy and Vernois found the pupils to be dilated after injecting belladonna into the cephalic vein, when the largest doses by the stomach had no effect. I would suggest that belladonna and stramonium are remedies which ought to do good in cholera—as they, more than any other substances, induce relaxation of contracted organic muscular fibres. They should be tried by inhalation, as has been done in asthma; and I venture to propose, also, what I believe has never been thought of—*warm baths of the infusion of stramonium leaves*, in the incipiency of the collapse. Anodyne baths are now frequently used for other purposes. Great success is reported, in a recent journal, to have attended the employment of hot *hop* baths in tetanus.

It is proper to try all imaginable remedies in a disease so desperate as cholera, with whose therapeia we are, at last, so imperfectly acquainted.

Belladonna, or atropia, might be injected, with a saline solution, into the bronchial or saphena vein: and quinine might be employed in a similar mode. It must be remembered, however, that, even used in this way, medicines do not have their ordinary effect in proportion to the dose, on account of the morbid state of the nervous centres. Thus, Magendie‡ found that two grains of camphor, diffused in water and injected into the veins of a cat, will make the animal bound several feet; but, in a case of collapse, when ʒss. of camphor was similarly injected, not the smallest excitement was produced.

In regard to *calomel*, there is no indication for its use to be drawn from the morbid anatomy, or from the inferred pathology

* Report of Dr. Gull to Royal Coll. of Phys., p. 209.
† Report of Dr. Gull, p. 215.
‡ London Med. and Surg. Journal, Vol. ii., p. 586.

of the disease. The argument in its favor, from the absence of bile in the stools, is rebutted by the fact of its abundance in the gall-bladder; and the clinical experience so commonly quoted on its behalf, is to be accounted for by the universal addition to it of opium in the prescription. In fact, however, the amount of success claimed for it is not very great, even with this adjunct. Such is the opinion of Dr. Gull, based upon the materials collected in his well-known and elaborate report.

I have but to remark, farther, upon the *rate of progress* of the disease. Phthisis may be a complaint of years; whooping-cough, of months; typhus, of weeks; pneumonia, of days; but cholera must be numbered by its hours, half-hours, or even *minutes*.

The rate of treatment, then, must be in proportion. We should give medicine, if we give it at all, in small quantities, every five or ten minutes: and especial perseverance must be maintained in the use of external applications, until the patient has fairly commenced to improve. When the blood begins to run through the veins of the hand so fast that the eye cannot follow it, after having been, as it often is, nearly at rest, he is mostly safe.

As to the third stage, or consecutive fever, of cholera, it is, perhaps, sufficient to say, that the treatment ought to be mild, palliative, expectant, and carefully supporting. Too little done, here, is better than too much.

If nothing new is added in the above short resumé of the treatment of cholera, my purpose is answered if the opinion be clearly conveyed, that a careful survey of the best recorded experience warrants the elimination of much that has been relied upon, especially in regard to the hepatic and constitutional medication of the collapsed stage.

